# Allelic Heterogeneity at the Equine *KIT* Locus in Dominant White (*W*) Horses

**DOI:** 10.1371/journal.pgen.0030195

**Published:** 2007-11-09

**Authors:** Bianca Haase, Samantha A Brooks, Angela Schlumbaum, Pedro J Azor, Ernest Bailey, Ferial Alaeddine, Meike Mevissen, Dominik Burger, Pierre-André Poncet, Stefan Rieder, Tosso Leeb

**Affiliations:** 1 Institute of Genetics, Vetsuisse Faculty, University of Berne, Berne, Switzerland; 2 DermFocus, Vetsuisse Faculty, University of Berne, Berne, Switzerland; 3 M. H. Gluck Equine Research Center, University of Kentucky, Lexington, Kentucky, United States of America; 4 Institute of Prehistory and Archaeological Sciences, University of Basel, Basel, Switzerland; 5 Department of Genetics, University of Cordoba, Gregory Mendel Building, Cordoba, Spain; 6 Division of Veterinary Pharmacology and Toxicology, Vetsuisse Faculty, University of Berne, Berne, Switzerland; 7 Swiss National Stud, Avenches, Switzerland; 8 Swiss College of Agriculture, Zollikofen, Switzerland; University of Oxford, United Kingdom

## Abstract

White coat color has been a highly valued trait in horses for at least 2,000 years. Dominant white (*W*) is one of several known depigmentation phenotypes in horses. It shows considerable phenotypic variation, ranging from ∼50% depigmented areas up to a completely white coat. In the horse, the four depigmentation phenotypes roan, sabino, tobiano, and dominant white were independently mapped to a chromosomal region on ECA 3 harboring the *KIT* gene. KIT plays an important role in melanoblast survival during embryonic development. We determined the sequence and genomic organization of the ∼82 kb equine *KIT* gene. A mutation analysis of all 21 *KIT* exons in white Franches-Montagnes Horses revealed a nonsense mutation in exon 15 (c.2151C>G, p.Y717X). We analyzed the *KIT* exons in horses characterized as dominant white from other populations and found three additional candidate causative mutations. Three almost completely white Arabians carried a different nonsense mutation in exon 4 (c.706A>T, p.K236X). Six Camarillo White Horses had a missense mutation in exon 12 (c.1805C>T, p.A602V), and five white Thoroughbreds had yet another missense mutation in exon 13 (c.1960G>A, p.G654R). Our results indicate that the dominant white color in Franches-Montagnes Horses is caused by a nonsense mutation in the *KIT* gene and that multiple independent mutations within this gene appear to be responsible for dominant white in several other modern horse populations.

## Author Summary

White horses have always been highly valued by their human owners. Their important role in history is reflected by their widespread use as heraldic animals (e.g., on the flags of the German states of Lower Saxony and North Rhine-Westphalia). In the Swiss Franches-Montagnes Horse population, a completely white mare named Cigale was born out of solid brown parents in 1957. The white phenotype is inherited as an autosomal dominant trait and all living white Franches-Montagnes Horses are descendants of Cigale. We sequenced the *KIT* gene in white and solid-colored Franches-Montagnes Horses and found a mutation that inactivates the gene product and thus leads to a lack of pigment-forming cells in the skin of white horses. We then analyzed white horses from other populations and found three additional independent candidate causative mutations in white Thoroughbreds, Arabians, and Camarillo White Horses. The research thus revealed independent mutation events leading to white coat color in different horse populations. Our findings will allow genetic testing and a more precise classification of horses with white coat color.

## Introduction

Dominant white (*W*) is an autosomal dominant trait that is characterized by coat depigmentation of variable extent. Caused by the absence of melanocytes from the depigmented skin areas, expressivity can range from ∼50% depigmented areas up to a nearly completely white coat. In contrast to horses with grey coat color (*G*), which are characterized by progressive greying of the hair, white horses show the depigmentation at birth and have a depigmented skin. Eyes are normally pigmented in dominant white horses, probably due to the different origin of the retinal melanocytes, which develop from local neuroectoderm and not from the neural crest, as do the skin melanocytes. In various horse breeds, cases of white or almost white horses born out of solid-colored parents have been reported [1]. Some breed registries have restrictions towards this phenotype, or do not allow the phenotype to be registered. In the Franches-Montagnes Horse population white horses are known and reported to trace back to the white founder mare Cigale, born in 1957 [2]. The Camarillo White Horses, which have a similar depigmentation phenotype, represent another famous line of horses that can be traced back to the white founder stallion Sultan, born in 1912 [3]. According to anecdotal reports from breeders, the dominant white phenotype appears to have originated independently on several occasions in Thoroughbreds. The white coat color phenotype is inherited as a monogenic autosomal dominant trait. In one study, white horses were shown to be obligate heterozygous (*W/+*), as the *W/W* genotype was hypothesized to cause early embryonal lethality [4].

In horses, the four depigmentation phenotypes roan, sabino-1, tobiano, and dominant white were independently mapped to a region on equine Chromosome 3 (ECA 3) harboring the *KIT* gene [2,5–7]. The sabino-1 spotting pattern is caused by an intronic mutation in the *KIT* gene, which causes partial skipping of exon 17 [6]. The tobiano spotting pattern is caused by a large chromosomal inversion that disrupts a potential regulatory element downstream of the *KIT* gene [7]. In contrast to sabino and tobiano, the mutations for roan and dominant white have been reported to cause lethality in the homozygous state in some horse breeds [4,8].

KIT is a type III receptor protein-tyrosine kinase and belongs to a protein subfamily including the colony stimulating factor-1 receptor (CSF1R), platelet-derived growth factor receptor (PDGFR), and fms-related tyrosine kinase 3 (FLT3). KIT contains an extracellular domain composed of five immunoglobulin domains, a single transmembrane domain, a juxtamembrane domain, and an intracellular protein kinase domain that is interrupted by an insertion of about 80 amino acids [9]. The KIT ligand (KITL), also called stem cell factor (SCF), binds to KIT via the second and third extracellular immunoglobulin domains. Ligand binding induces receptor dimerization, thereby activating the intrinsic tyrosine kinase domain through transphosphorylation and further signal transduction [10]. KIT has the potential to participate in multiple signaling pathways, which accounts for its important role in the control of cell differentiation, proliferation, survival, and motility. A complete loss of function of KIT causes prenatal or perinatal lethality due to anemia [11]. KIT signaling is crucial for the development and survival of melanoblasts, mast cells, spermatogonia, and the interstitial cells of Cajal in the gastrointestinal tract [12]. Receptor-inactivating point mutations in the *KIT* gene often act dominantly or semidominantly and are associated with hypopigmentation, anemia, and/or sterility. The dominant or semidominant inheritance is either due to haploinsufficiency or to the fact that mutated KIT receptors may form heterodimers with wild-type KIT receptors and thus act fully or partially dominant negative. Inactivating KIT mutations cause piebaldism in humans and white or spotted *W* mutants in mice [13–17]. Gain-of-function mutations in the proto-oncogene KIT are involved in the formation of gastrointestinal stromal tumors, myelogenous leukemia, and mastocytomas [18].

Here, we report the complete genomic organization of the equine *KIT* gene and an analysis of the *KIT* gene sequence in horses with depigmentation phenotypes.

## Results

### Genomic Sequence of the Equine *KIT* Gene

We identified and sequenced an equine BAC clone harboring the entire *KIT* gene. This sequence corresponds to positions 6,812,854–7,023,775 on scaffold 10 of the first horse genome assembly, which was published during the progress of our work. The genomic organization of the equine *KIT* gene was inferred by comparison of the genomic sequence with the cDNA sequence from public databases. The equine *KIT* gene spans a genomic region of about 82 kb and comprises 21 exons, similar to the human *KIT* gene. The equine *KIT* mRNA contains an open reading frame of 2,919 bp encoding a protein of 972 amino acids. The encoded peptide is predicted to have a molecular weight of 108.9 kDa, a pI of 6.3, and 88% identity to the human and 82% identity to the orthologous mouse protein, respectively.

### Mutation Analysis in Franches-Montagnes Horses

**Figure 1:**
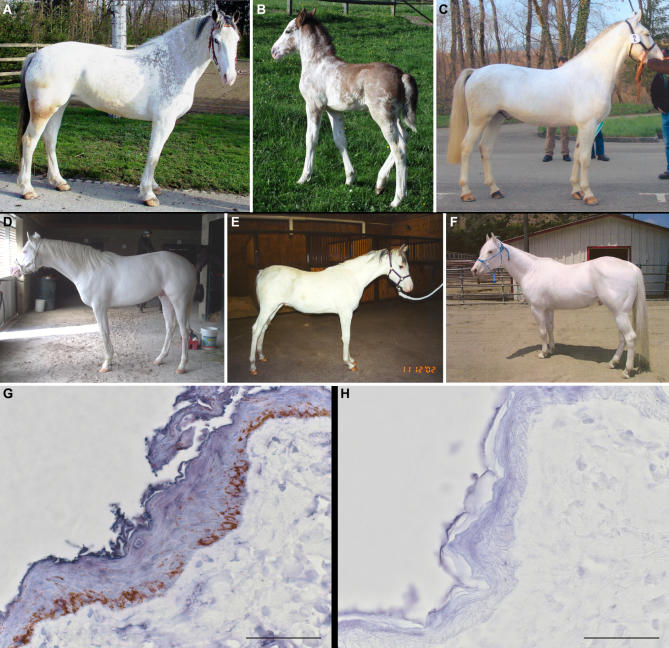
Dominant White Phenotype in Horses (A) Franches-Montagnes mare with little residual pigmentation. (B, C) Dominant white Franches-Montagnes stallion showing partial depigmentation as a colt and almost complete depigmentation at 4 years of age. (D) Dominant white Thoroughbred stallion. (E) Dominant white Arabian stallion. (F) Camarillo White Horse. (G) Immunohistochemistry using a polyclonal KIT antibody on a skin biopsy from a solid-colored horse. Blue staining indicates KIT expression throughout the epidermis. Melanin produced by melanocytes is visible as brown granules. (H) Immunohistochemistry on a skin biopsy of a white horse. Note the weak blue staining and the complete absence of melanocytes and melanin. The bars correspond to 50 μm.

**Figure 2:**
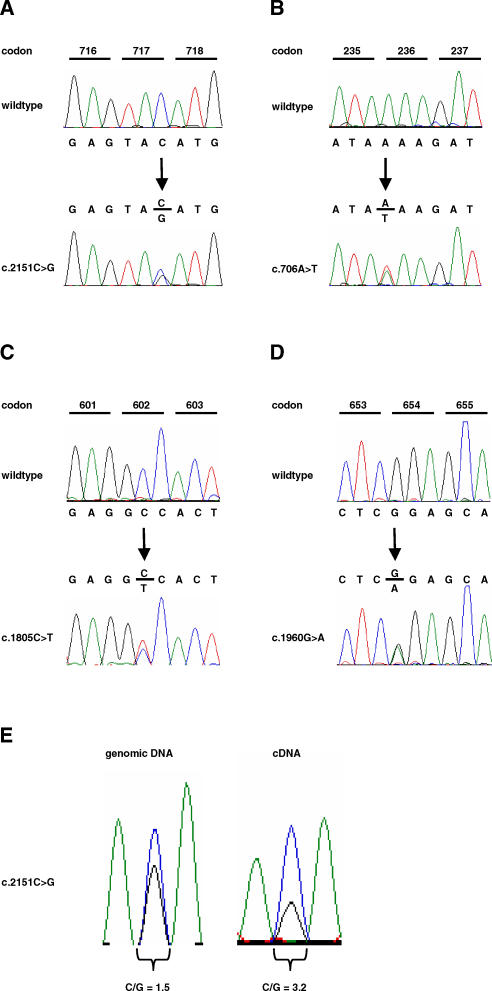
Mutations in the Equine *KIT* Gene Direct sequencing of genomic PCR products from solid-colored and dominant white horses revealed four nonsynonymous polymorphisms that were exclusively found in white horses. (A) Exon 15, c.2151C>G mutation in a white Franches-Montagnes Horse. (B) Exon 4, c.706A>T mutation in a white Arabian. (C) Exon 12, c.1805C>T mutation in a Camarillo White Horse. (D) Exon 13, c.1960G>A mutation in a White Thoroughbred. (E) Electropherograms of exon 15 sequences from a dominant white Franches-Montagnes Horse derived from genomic DNA and cDNA. The presence of the polymorphism is clearly visible on the genomic DNA and on the transcript level. The reduced signal intensity of the mutant G allele in the cDNA sequence indicates a reduction of the mutant transcripts compared to the wild-type transcripts of about 50%.

We selected four white and four solid-colored Franches-Montagnes horses from a three-generation family for the initial mutation analysis (see Figure 1 for an overview on phenotypes and Figure S1 for pedigree of the family). Comparative sequencing of all 21 exons and adjacent sequences revealed 15 SNPs in these closely related animals (Table 1). Only one of the 15 polymorphisms showed perfect cosegregation of the genotypes with the dominant white phenotype in the initial family. The cosegregating polymorphism was a C-to-G transversion located in exon 15 (c.2151C>G), which introduced a premature stop codon in the open reading frame of the KIT protein (p.Y717X). The mutation was predicted to truncate the KIT protein in the middle of the kinase insert domain (Figure S2). We confirmed the presence of the c.2151C>G polymorphism in the white horses by sequencing genomic PCR products containing exon 15 in both directions (Figure 2A) and performing BccI RFLP analysis of these PCR products. Furthermore, the polymorphism was also confirmed on the transcript level by sequencing reverse transcriptase PCR (RT-PCR) products (Figure 2E).

**Table 1:**
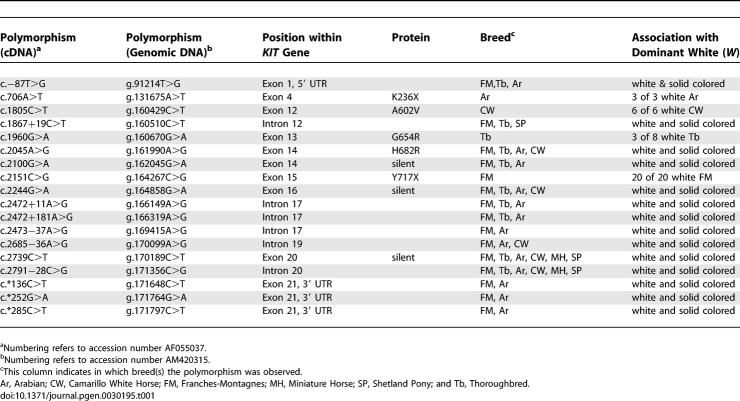
*KIT* Gene Polymorphisms

### Confirmation of the Association on the Population Level

We genotyped all available Franches-Montagnes samples (*n* = 132) in our laboratory for the c.2151C>G polymorphism. All 20 white horses, descendants of Cigale, were heterozygous for the c.2151C>G polymorphism (Figure S1). To exclude the possibility that this variant also occurs in solid-colored horses, a cohort of 112 solid-colored Franches-Montagnes Horses were genotyped and all of them were homozygous for the wildtype C allele at position c.2151 (Table S1).

### Functional Analysis of the c.2151C>G Mutation

Transcripts containing premature termination codons are often subject to nonsense-mediated decay (NMD) or nonsense-mediated exon skipping (NME). NMD and NME are thought to be quality-control mechanisms by which cells can limit the expression of aberrant proteins. We performed RT-PCR on leukocyte RNA from a white horse using primers located in exon 13 and exon 16. Analysis of the RT-PCR products on agarose gels exclusively yielded the complete cDNA-fragments including exon 15. Therefore, the c.2151C>G mutation does not seem to induce NME (unpublished data).

Direct sequencing of the RT-PCR product from a heterozygous white horse revealed that the mutant transcript is present, albeit at a lower level compared to the wild-type transcript. From the quantitative analysis of the signal intensity ratios in the electropherograms between genomic DNA and cDNA, we estimated that the amount of mutant transcript is about 50% of the amount of wild-type transcript in leukocytes (Figure 2C). The other 50% of mutant transcripts are probably degraded by NMD.

**Figure 3:**
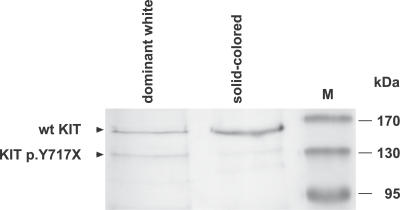
Western Blot Analysis of KIT Protein Expression in Skin Samples A strong band of the expected size of glycosylated wild-type KIT protein was detected in the protein sample from the solid-colored horse. Protein extract from the skin of a dominant white horse yielded two very weak bands. The sizes of the two bands corresponded to wild-type and truncated KIT protein, respectively. As the skin sample from the dominant white horse did not contain melanocytes, the weak bands presumably were due to other KIT-expressing cells that may have been present in the skin biopsy.

We further performed a western blot on protein extracts from skin samples of a dominant white and a solid-colored horse to investigate whether the predicted truncated KIT protein is expressed in skin. The western blot of the solid-colored horse showed a strong band of the expected size (∼150 kDa) for the glycosylated full-length KIT protein. The skin sample from the dominant white horse yielded a weak band at ∼150 kDa and another weak band at ∼120 kDa. The size of the ∼120 kDa band corresponds to the predicted size of the truncated KIT protein, as the p.Y717X mutation should remove 29 kDa of the presumably unglycosylated intracellular part of the protein (Figure 3).

### Analysis of White Horses from other Breeds

We sequenced all 21 *KIT* exons in 16 other white or partially depigmented horses from other breeds (Arabian, Camarillo White Horses, Thoroughbred, Miniature Horse, Shetland Pony). Eight of these 16 horses carried nonsynonymous mutations in the *KIT* coding sequence. Two related white Arabians had a nonsense mutation in exon 4 (c.706A>T, Figure 2B), which was predicted to truncate the protein in the extracellular domain (p.K236X). A third Arabian horse in the family with minor white spotting did not have the mutation. The mutation showed perfect cosegregation with the dominant white phenotype in the family of the tested Arabians (Figure S1). It was absent from 20 unrelated solid-colored Arabians and also from 110 solid-colored Franches-Montagnes Horses that were genotyped as controls (Table S1).

Three Camarillo White Horses were heterozygous for a missense mutation in exon 12 (c.1805C>T, Figure 2C). This mutation affects the intracellular tyrosine kinase domain of the KIT protein and replaces the small side chain of alanine 602 with the much larger side chain of a valine (p.A602V). The mutation was exclusively found in the white horses from the family of the tested Camarillo White Horses (Figure S1). Camarillo White Horses are an open breed and can be mated to horses from other populations without restrictions. However, only white offspring of such matings can be registered as a Camarillo White Horse. Therefore, it was not possible to analyze solid-colored horses from the same registry as controls. However, the mutation was absent from 169 solid-colored horses of various breeds (Table S1).

Three related white Thoroughbreds were heterozygous for a missense mutation in exon 13 (c.1960G>A, Figure 2D). This mutation also affects the intracellular tyrosine kinase domain of the KIT protein and leads to a nonconservative exchange of glycine-654 with arginine (p.G654R). The cosegregation of the mutation was confirmed in the family of the tested animals (Figure S1). This mutation was absent from 17 solid-colored Thoroughbreds and 97 solid-colored Franches-Montagnes Horses (Table S1).

The presence of the c.1805C>T and c.1960G>A polymorphisms was independently confirmed by RFLP analyses. We did not find any mutations affecting the *KIT* coding sequence in the remaining eight horses with depigmentation phenotypes. These horses were five almost completely white Thoroughbreds, one Arabian Horse with minor white spotting, one Miniature Horse with ∼50 % depigmented skin area, and one Shetland Pony with ∼75% depigmented skin area.

## Discussion

The data of this study strongly suggest that the c.2151C>G mutation causes the dominant white phenotype segregating in the Franches-Montagnes Horses. As the mutation represents a nonsense mutation, which leads to the truncation of the functionally important second half of the intracellular tyrosine kinase domain of the KIT protein, it seems justified to assume that this mutation severely affects KIT function. Furthermore, the perfect association of this mutation with the dominant white phenotype in cohorts of 20 white and 112 solid-colored Franches-Montagnes Horses corroborates the causative role of the c.2151C>G mutation. The observed association would also be compatible with a scenario of a closely adjacent causative mutation, which is in linkage disequilibrium with the c.2151C>G mutation. However, we regard this as very unlikely, as we can rule out any mutations in the other exons of the *KIT* gene. Therefore, it would be difficult to postulate a different causative mutation with a more dramatic effect on the KIT protein than the c.2151C>G mutation, which causes the truncation of more than a quarter of the protein, including the entire second intracellular tyrosine kinase domain.

A similar argument holds true for the c.706A>T mutation, which segregated in a family of white Arabians. This mutation is predicted to truncate more than three quarters of the protein, including ligand binding domain, transmembrane domain, and the entire intracellular part of the KIT protein.

The prediction of the functional impact of the two missense mutations found in Thoroughbreds and Camarillo White Horses is not quite as straightforward. However, both mutations affect the functionally important first intracellular tyrosine kinase domain, and comparable mutations of this domain have been shown to cause piebaldism in humans and depigmentation phenotypes in mice (Figure S2).

The c.2151C>G mutation was confirmed on the genomic and on the cDNA level. Apparently, only about half of the mutant *KIT* mRNA is cleared by NMD, although it does contain a premature stop codon. Investigation of the mutant protein expression in skin samples of a dominant white (*W/+*) and a solid-colored (*+/+*) horse by western blot showed a strong band of the expected size in the solid-colored (*+/+*) horse and weak bands corresponding to the sizes of the wild-type and the truncated protein in a dominant white (*W/+*) horse. Thus, the western blot indicated that the truncated protein is indeed expressed. It seems conceivable that a truncated KIT protein lacking the second half of the intracellular tyrosine kinase domain forms inactive dimers with wild-type KIT proteins and acts as a dominant-negative protein. The observed variation in the coat color phenotypes of horses with the c.2151C>G mutation (Figure 1A–1C) could be explained by different efficacies of NMD in different individuals and in different body regions. If the mutant transcript is efficiently cleared by NMD, then the remaining wild-type allele could possibly produce enough functional KIT protein to facilitate pigmentation to some extent. However, the more that the truncated KIT protein is expressed, the less likely it is that enough functional dimers of wild-type KIT proteins can mediate proper KIT signaling.

Our data indicate allelic heterogeneity among dominant white horses from different breeds. Thus, our study represents another example where different mutations in a single gene have been described for a Mendelian trait in domestic animals similar to the situation in, e.g., brown coat color in dogs or syndactyly in cattle [19,20]. At this time, it is not known whether the depigmentation of the other reported white horses is caused by as-yet undescribed mutations at the *KIT* locus or whether mutations in other genes can also cause this phenotype. The striking phenotype and the autosomal dominant inheritance facilitate the identification of founder animals. In each of the four investigated families, the white founder animals born out of solid-colored parents are known: for the Franches-Montagnes Horses, it is the white mare Cigale, born in 1957. In the Arabian family, the presumed founder stallion was born in 1996. The white stallion Sultan, born in 1912, is the reported founder of the Camarillo White Horses. The white founder animal of the Thoroughbred family segregating the c.1960G>A mutation is most likely a stallion born in 1946. In line with the recent origin of these mutation events, the four proposed candidate causative mutations of this study segregate only within the four respective families. In contrast, all of the other 14 *KIT* polymorphisms that were discovered during the initial mutation analysis segregate in at least two distinct horse populations, indicating that they are much older and spread into different horse populations by the ongoing admixture, which is typical for many modern horse breeds.

*KIT* gene mutations have been described in humans with piebaldism [13–15], in *W* mouse mutants [16,17], and in dominant white pigs [21]. White spotting or white coat color is a common trait in many breeds of domestic animals, but in many instances the molecular mechanisms for these depigmentation phenotypes are still unknown. In the horse, a mutation in the *EDNRB* gene encoding the endothelin receptor B causes the overo spotting pattern in the heterozygous state. When this mutation is present in the homozygous state, completely depigmented foals are born that usually die within the first few days of life, due to intestinal aganglionosis [22]. The equine *KIT* gene plays a central role in equine pigmentation, as at least four distinct depigmentation phenotypes are known to be associated with mutations at the equine *KIT* locus. Of the equine *KIT* mutations, so far, only the mutations for sabino-1 and tobiano have been elucidated at the molecular level. The mutations for the sabino-1 and tobiano spotting patterns do not change the *KIT* coding sequence, but rather reduce the expression of functional *KIT* transcripts. Tobiano (*TO*) acts strictly autosomal dominant, and *TO/TO* horses are viable, fertile, and phenotypically indistinguishable from *TO/+* horses [7]. Sabino-1 (*SB1*) acts semidominant, and *SB1/SB1* horses are viable and almost completely white, whereas *SB1/+* horses show a characteristic sabino spotting pattern [6]. Dominant white (*W*) produces a variable but rather severe depigmentation phenotype in heterozygous horses (*W/+*). In the mouse, more than 90 different *Kit* alleles are known. Many of these mutations produce some degree of white spotting in the heterozygous state, which can range from tiny white belly spots to >99% depigmentation. Many *KIT* regulatory mutations exist that produce severe pigmentation phenotypes in the heterozygous state. However, some of the murine structural mutations, such as the *Kit^W-42J^* mutation, also lead to an almost complete depigmentation in the heterozygous state. In the mouse, mutations causing a pronounced dominant depigmentation phenotype typically also lead to mild anemia and reduced male fertility even in the heterozygous state [23]. We are not aware of any specific health problems in the studied white horses. At least two of the white Thoroughbreds with the c.1960G>A mutation successfully competed in horse races, indicating a very good general fitness. There are very little data on fertility in white horses; however, one white Franches-Montagnes stallion was successfully used for artificial insemination, and all routine sperm parameters from this stallion were normal. Therefore, from the limited available data, it appears that heterozygous *KIT* mutations may have less detrimental effects in horses on hematopoiesis and fertility than in mice. In horses, dominant white was reported to cause embryonic lethality in the homozygous state [4]. However, this report on the embryonic lethality was derived from the analysis of offspring phenotype ratios in a single herd segregating one or more unknown mutations. As there is now evidence for allelic heterogeneity, it remains to be proven whether all equine dominant white mutations cause embryonic lethality in the homozygous state. While this is certainly likely for the two nonsense mutations found in Franches-Montagnes Horses and Arabians, it should not necessarily be assumed for the two reported missense mutations or for any of the other as-yet unknown *W* mutations.

White horses have always fascinated their human owners. The majority of “white” horses probably carry the greying-with-age mutation [24], which means that they are born solid colored and become white at the age of four to six years. However, there are also a number of historical reports that explicitly mention white-born horses resembling the phenotype of dominant white horses [1,25]. Two thousand years ago, the Romans already knew of the phenotypic differences of depigmented horses, which they described as candidus (white) or glaucus (grey) [26]. The Roman historian Tacitus described the use of sacred white horses for auguries by German tribes [27]. The so-called white horse of the Saxons is depicted on the flags of the German states of Lower Saxony and North Rhine-Westphalia. It is thus of considerable historic interest to trace the origins of white horses, particularly because the nature of their white color can have different causes, some of which are *KIT* mutations such as those described here. We do not know whether the Roman terms candidus and glaucus actually correspond to our modern coat color designations of white and grey. Archaeogenetics on historic DNA samples may help to identify the genetic constitution of these horses.

In conclusion, we have identified the probable causative mutation for the dominant white phenotype in Franches-Montagnes Horses. We have also identified three additional candidate causative mutations in Arabians, Camarillo White Horses, and Thoroughbreds. The knowledge of these mutations will allow genetic testing and should help to assign more precise coat color descriptions for partially or completely depigmented horses.

## Materials and Methods

### Animals.

All 21 KIT exons were analyzed in 138 horses of six different breeds. The animals consisted of 118 solid-colored horses (112 Franches-Montagnes, six Arabian), four white Franches-Montagnes, and 16 horses registered as maximal sabino (eight Thoroughbreds from three independent families, three related Arabians, three related Camarillo White Horses, one Miniature Horse, and one Shetland Pony). These 16 horses had been typed for the absence of the sabino-1- and tobiano-causing mutations [6,7]. In the Franches-Montagnes breed, horses with spotting phenotypes (sabino, tobiano, and overo) may not be registered; therefore, we assumed these mutations to be absent from the Franches-Montagnes population. Sixteen additional white Franches-Montagnes Horses were genotyped for the presence of the c.2151C>G mutation.

Additional solid-colored animals from various breeds (Arabian, Missouri Fox Trotter, Quarter Horse, Spanish Mustang, Spotted Draft, Spotted Mountain Horse, and Thoroughbred) were tested for the absence of the four candidate causative mutations.

### DNA and RNA extraction.

Genomic DNA was isolated from peripheral blood or hair roots using standard methods. RNeasy spin columns were used to isolate total RNA from a solid-colored horse (skin, small intestine, colon, and testis) and a white horse (skin) according to the manufacturer's protocol (Qiagen). Additionally, total RNA was isolated from white blood cells of the white horse using TRIzol reagent (Invitrogen) according to the manufacturer's protocol.

### DNA sequencing.

The BAC clone CH241-440E11 from the CHORI-241 library was predicted to contain the equine *KIT* gene, based on BAC end sequence comparative mapping [28]. DNA from this clone was isolated using the Qiagen large construct kit (Qiagen). The purified BAC DNA was sheared to approximately 2–5 kb fragments using a nebulizer, and a shotgun plasmid library was prepared in the vector pCR4Blunt-TOPO (Invitrogen). Template DNA for sequencing was prepared using TempliPhi (GE Healthcare) and shotgun plasmid clones were sequenced to approximately 6-fold coverage using the BigDye v3.1 kit and an ABI 3730 capillary sequencer (Applied Biosystems). After sequencing a random collection of plasmid subclones, the remaining gaps were closed by a primer walking strategy. Sequence data were assembled with Sequencher 4.6 (GeneCodes). Comparison of the obtained genomic DNA sequence with a published *KIT* cDNA sequence (AJ224645) allowed the annotation of the exons.

### PCR and mutation analysis.

Primers for the amplification of each of the 21 KIT exons with flanking regions were designed with the software Primer3 (http://frodo.wi.mit.edu/cgi-bin/primer3/primer3_www.cgi) after masking repetitive sequences with RepeatMasker (A. F. A. Smit and P. Green, http://repeatmasker.genome.washington.edu/). The sequences of the primers are listed in Table S2. PCR products were amplified in 20 μl reactions using AmpliTaq Gold DNA polymerase (Applied Biosystems) and checked for yield and purity on agarose gels. Direct sequencing of the PCR products was performed after shrimp alkaline phosphatase (Roche) and exonuclease I treatment (New England Biolabs). PCR products were sequenced as described above using both PCR primers as sequencing primers. In some instances, additional internal sequencing primers were used.

### RFLP analysis.

PCR products were subjected to RFLP analyses to confirm the presence of three nonsynonymous mutations. The presence of the c.1805C>T mutation was verified using the enzyme HaeIII. The c.1960G>A mutation was verified with DdeI, and the c.2151C>G mutation with BccI. The restriction fragments were separated on standard agarose gels and genotypes were determined from the resulting band patterns.

### RT-PCR.

Aliquots of 1 μg total RNA were reverse transcribed into cDNA using 20 pmol (T)_24_V primer and Omniscript reverse transcriptase (Qiagen). Two microliters of the cDNA were used as a template in PCR. PCRs were performed as described above.

### Immunohistochemistry.

Epidermis from a solid-colored and a white horse was fixed in formalin-buffered saline and embedded in paraffin. Tissue sections (5 mm) were dewaxed in xylene for 7 min, dehydrated in alcohol, and then rinsed with PBS. For exposure and detection of KIT protein, antigen retrieval was performed in Tris-EDTA (pH 9) in the microwave for 15 min. Nonspecific binding was blocked by incubating the sections in 5% normal donkey serum (Dako) in Tris-buffered saline for 30 min. Negative control studies were performed without primary antibody. We used a goat antibody directed against the extracellular domain of mouse c-kit (R&D Systems) at a concentration of 2 μg/ml. Binding was detected using an alkaline phosphatase donkey-anti-goat IgG (Jackson Immuno Research) at a dilution of 1:200 for 60 min. Sections were washed in PBS and subsequently visualized using BCIP/NBT (Dako).

### Western blot.

For skin protein extraction, minced skin samples were incubated overnight at 4 °C with RIPA extraction buffer (Pierce) containing 25 mM Tris-HCl pH 7.6, 150 mM NaCl, 1% NP-40, 1% sodium deoxycholate, and 0.1% SDS, complemented with Halt protease inhibitor cocktail (Pierce) and 8 M urea. After centrifugation at 15,000 xg for 10 min at 4 °C, the supernatants were collected and protein concentration was determined using a Bradford assay (Bio-Rad). Protein extracts (20 μg) were boiled for 5 min in reducing sample buffer and separated by 12% SDS-PAGE. Proteins were electrically transferred to a polyvinylidene difluoride (PVDF) membrane. After blocking with 5% skim milk, the membrane was incubated with a chicken IgY affinity-purified polyclonal antibody directed against the N-terminus of human KIT (GenWay) diluted 1:1,000. Detection was performed using an alkaline phosphatase–conjugated anti-IgY secondary antibody (Jackson Immuno Research) diluted 1:5,000 and BCIP/NBT (Dako).

## Supporting Information

Figure S1 Pedigrees of White Horses (31 KB PDF)

Figure S2 Multiple Alignment of the Equine, Human, and Murine KIT proteins (24 KB PDF)

Table S1 Genotype Distributions at *KIT* Polymorphisms (18 KB PDF)

Table S2 Primer Sequences for the Amplification of Equine *KIT* Exons and cDNA Fragments (12 KB PDF)

### Accession Numbers

The DNA sequence reported in this manuscript has been submitted to the European Molecular Biology Laboratory (EMBL) database (http://www.ebi.ac.uk/embl/) under accession number AM420315.

The National Center for Biotechnology Information (NCBI) Entrez database (http://www.ncbi.nlm.nih.gov/sites/gquery) accession number for scaffold 10 of the first horse genome assembly is NW_001799714, and the accession for a partial equine *KIT* mRNA sequence is AF055037. This sequence was used as a reference for numbering the positions of the sequence variants.

## Abbreviations

NMD: nonsense-mediated decay
NME: nonsense-mediated exon skipping
RT-PCR: reverse transcriptase PCR
