# Supplementary material for: Allelic Heterogeneity at the Equine KIT Locus in Dominant White (W) Horses
Source: PLoS Genet. 2007 Nov 9;3(11):e195. doi: 10.1371/journal.pgen.0030195 (PMC2065884; doi:10.1371/journal.pgen.0030195)
Supplement: Supplementary file 1 [file pgen.0030195.sg001.pdf]

Figure S1. Pedigrees of white horses. All known white Franches-Montagnes Horses trace back to the founder Cigale born in 1957. In the Franches-Montagnes Horses genotypes at the c.2151C>G mutation are indicated for 20 white and four solid-colored horses that were available for genotyping. The numbers of the eight horses used for the initial mutation analysis are underlined. Note that some stallions appear twice in the pedigree. The resulting loops were not drawn to keep the pedigree concise. In the Camarillo White Horse family 15 animals were available for genotyping and the genotypes at the c.1805C>T mutation are indicated. Two horses from the American White registry with a probably unrelated depigmentation phenotype are represented by grey filled symbols. In the Arabian family six animals were available for genotyping and the genotypes at the c.706A>T mutation are indicated. In the Thoroughbred family six horses were available for genotyping and the genotypes at the c.1960G>A mutation are indicated.

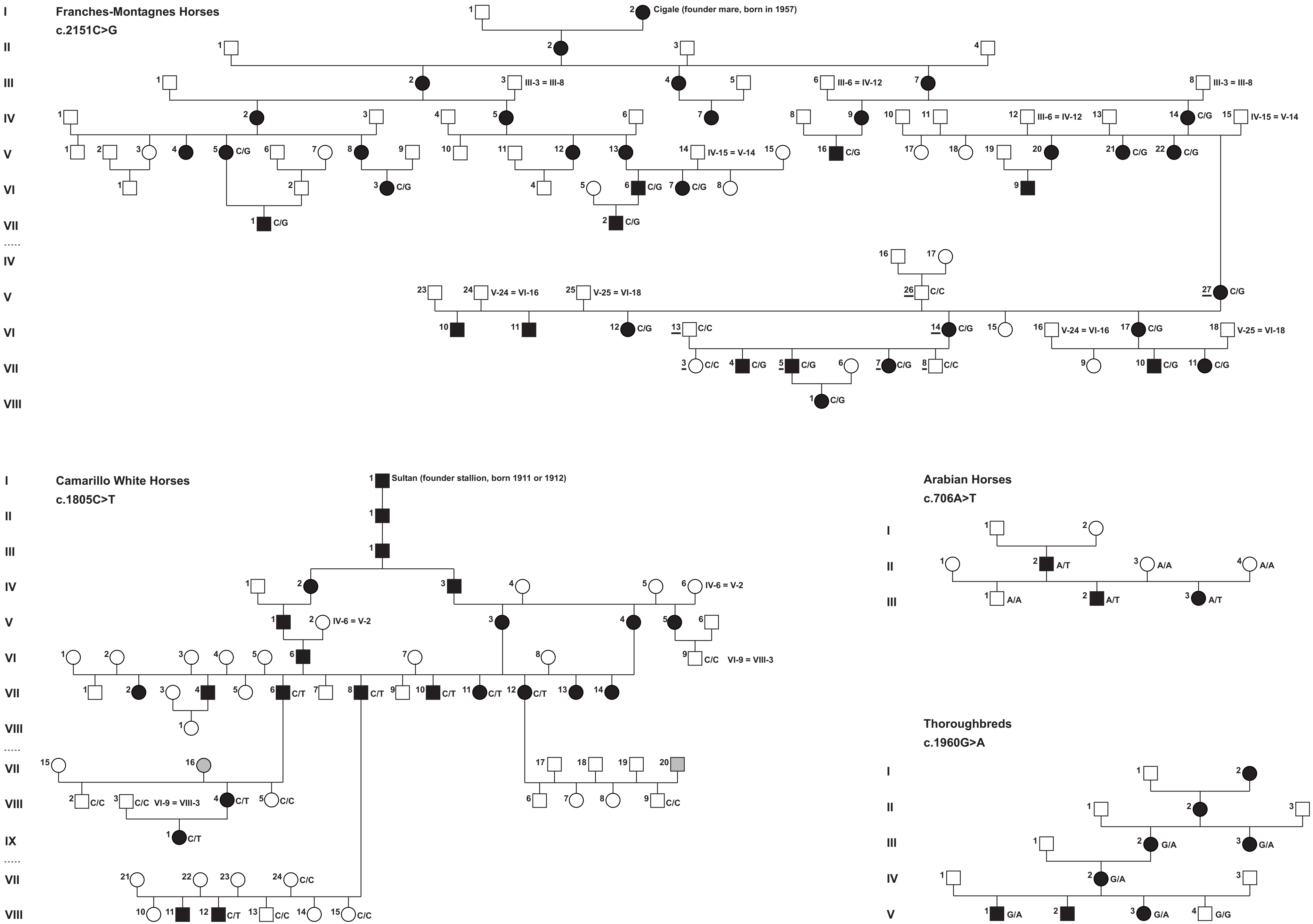

Franches-Montagnes Horses
c.2151C>G
Cigale (founder mare, born in 1957)
III-3 = III-8
III-6 = IV-12
IV-15 = V-14
V-24 = VI-16
V-25 = VI-18
Camarillo White Horses
c.1805C>T
Sultan (founder stallion, born 1911 or 1912)
IV-6 = V-2
VI-9 = VIII-3
Arabian Horses
c.706A>T
Thoroughbreds
c.1960G>A
