# Supplementary material for: Allelic Heterogeneity at the Equine KIT Locus in Dominant White (W) Horses
Source: PLoS Genet. 2007 Nov 9;3(11):e195. doi: 10.1371/journal.pgen.0030195 (PMC2065884; doi:10.1371/journal.pgen.0030195)
Supplement: Supplementary file 2 [file pgen.0030195.sg002.pdf]

Figure S2. Multiple alignment of the equine, human and murine KIT proteins. The sequences were derived from Genbank accessions AM420315 (horse), NP_000213 (human), and NP_066922 (mouse). Individual protein domains are indicated by colored shading. The position of selected known human and murine KIT mutations is shown. The four newly described equine KIT mutations are highlighted in grey.

```
HORSE           MRGARGAWDFLCVLLLLFRVQTGSSQPSVSPGELSPPSIHPAKSELIVSVGDEIRLLCAD 60
HUMAN           MRGARGAWDFLCVLLLLLRVQTGSSQPSVSPGEPSPPSIHPGKSDLIVRVGDEIRLLCTD 60
MOUSE           MRGARGAWDLLCVLLVLLRGQTATSQPSASPGEPSPPSIHPAQSELIVEAGDTLSLTCID 60
                <1-22 signal peptide >

                          ↓ K86Rfs17X (human piebaldism)
HORSE           PGFVKWTFET-WGQLSENTHKEWVTEKAEATNTGSYTCTNEGGLSSSIYVFVRDPAKLFL 119
HUMAN           PGFVKWTFEI-LDETNENKQNEWITEKAEATNTGKYTCTNKHGLSNSIYVFVRDPAKLFL 119
MOUSE           PDFVRWTFKTYFNEMVENKKNEWIQEKAEATRTGTYTCSNSNGLTSSIYVFVRDPAKLFL 120

HORSE           FDPSLYGKEGSDTLVRCPLTDPEVTNYSLMACEGKSLPKDLTFVADPKAGITIRNVKREY 179
HUMAN           VDRSLYGKEDNDTLVRCPLTDPEVTNYSLKGCQGKPLPKDLRFIPDPKAGIMIKSVKRAY 179
MOUSE           VGLPLFGKEDSDALVRCPLTDPQVSNYSLIECDGKSLPTDLTFVPNPKAGITIKNVKRAY 180

                                                        ↓ K236X (dominant white, Arabian Horses)
HORSE           HRLCLRCSADKDGKSVLSNKFTLKVRAAIRAVPVVSVSKASYLLREGEEFSVTCLIKDVS 239
HUMAN           HRLCLHCSVDQEGKSVLSEKFILKVRPAFKAVPVVSVSKASYLLREGEEFTVTCTIKDVS 239
MOUSE           HRLCVRCAAQRDGTWLHSDKFTLKVREAIKAIPVVSVPETSHLLKKGDTFTVVCTIKDVS 240

HORSE           SSVDSMWIRENSR--TKEQVKSSSSHQGDFNFVRQERLTISPARVNDSGVFMCYANNTFG 297
HUMAN           SSVYSTWKRENSQ--TKLQEKYNSWHHGDFNYERQATLTISSARVNDSGVFMCYANNTFG 297
MOUSE           TSVNSMWLKMNPQPQHIAQVKHNSWHRGDFNYERQETLTISSARVDDSGVFMCYANNTFG 300

HORSE           SANVTTTLEVVDKGFINVFPMMNTTVFVNDGENVDLIVEYESYPKPEHQQWIYMNRTSTD 357
HUMAN           SANVTTTLEVVDKGFINIFPMINTTVFVNDGENVDLIVEYEAFPKPEHQQWIYMNRTFTD 357
MOUSE           SANVTTTLKVVEKGFINISPVKNTTVFVTDGENVDLVVEYEAYPKPEHQQWIYMNRTSAN 360

                                                 ↓ V410F (mouse, Kit^{W-2Bao}, semidominant white)
HORSE           KWEDYPKSENESNIRYVSELHLTRLKGTEGGTYTFLVSNSDVDSSVTFNVYVNTKPEILT 417
HUMAN           KWEDYPKSENESNIRYVSELHLTRLKGTEGGTYTFLVSNSDVNAAIAFNVYVNTKPEILT 417
MOUSE           KGKDYVKSDNKSNIRYVNQLRLTRLKGTEGGTYTFLVSNSDASASVTFNVYVNTKPEILT 420

HORSE           RDRLMNGMLQCVAAGFPEPTIDWYFCPGTEQRCSIPVGPVDVKIQNSSVSPFGKLVVQSS 477
HUMAN           YDRLVNGMLQCVAAGFPEPTIDWYFCPGTEQRCSASVLPVDVQTLNSSGPPFGKLVVQSS 477
MOUSE           YDRLINGMLQCVAEGFPEPTIDWYFCTGAEQRCTTPVSPVDVQVQNVSVSPFGKLVVQSS 480

HORSE           IDYSAFKHNGTVECRAYNDVGKSSAFFNFAFK----EQIHPHTLFTPLLIGFVVAAGMMC 533
HUMAN           IDSSAFKHNGTVECKAYNDVGKTSAYFNFAFKGNNKEQIHPHTLFTPLLIGFVIVAGMMC 537
MOUSE           IDSSVFRHNGTVECKASNDVGKSSAFFNFAFK----EQIQAHTLFTPLLIGFVVAAGAMG 536
                                                           <521-543 transmembrane domain

                                                                          ↓ G595R (mouse Kit^{W-Jic}, semidominant white spotting)
                                                             ↓ E582K (mouse Kit^{W-37J}, semidominant white spotting)
                                                ↓ Y569F (mouse Kit^{tm2Ber}, dominant extreme white spotting)
                                              ↓ Y567F (mouse Kit^{tm1Ber}, dominant extreme white spotting)

                                                             ↓ E584K (human piebaldism)
                   ↓ M541L             ↓ E561Gfs18X           ↓ F584L & F584C (4x human piebaldism)
HORSE           VIVMVLTYKYLQKPMYEVQWKVVEEINGNNYVYIDPTQLPYDHKWEFPRNRLSFGKTLGA 593
HUMAN           IIVMILTYKYLQKPMYEVQWKVVEEINGNNYVYIDPTQLPYDHKWEFPRNRLSFGKTLGA 597
MOUSE           IIVMVLTYKYLQKPMYEVQWKVVEEINGNNYVYIDPTQLPYDHKWEFPRNRLSFGKTLGA 596
 transmembrane domain><    544-581 juxtamembrane domain    ><582-937 protein kinase domain

                ↓ G597A (mouse Kit^{W-ei}, dominant white)      ↓ K641E (mouse Kit^{tm1Bpr}, recessive white)

                                       ↓ A621S              ↓ K642Sfs6X (2x human piebaldism)
                          ↓ A602V (dominant white, Camarillo White Horses)
HORSE           GAFGKVVEATAYGLIKSDAAMTVAVKMLKPSAHLTEREALMSELKVLSYLGNHMNIVNLL 653
HUMAN           GAFGKVVEATAYGLIKSDAAMTVAVKMLKPSAHLTEREALMSELKVLSYLGNHMNIVNLL 657
MOUSE           GAFGKVVEATAYGLIKSDAAMTVAVKMLKPSAHLTEREALMSELKVLSYLGNHMNIVNLL 656

                   ↓ T660M (mouse Kit^{W-v}, semidominant white spotting)
                      ↓ G664R (human piebaldism)
                ↓ G654R (dominant white, Thoroughbreds)
HORSE           GACTVGGPTLVITEYCCYGDLLNFLRRKHDSFICSKQEDHAEAALYKNLLHSKESSCNDS 713
HUMAN           GACTIGGPTLVITEYCCYGDLLNFLRRKRDSFICSKQEDHAEAALYKNLLHSKESSCSDS 717
MOUSE           GACTVGGPTLVITEYCCYGDLLNFLRRKRDSFIFSKQEEQAEAALYKNLLHSTEPSC-DS 715
                   protein kinase domain  ><  685-761 kinase insert domain

                   ↓ Y719F (mouse Kit^{tm1.1Bsm}, infertility, no pigmentation phenotype)
                   ↓ Y717X (dominant white, Franches-Montagnes Horses)
HORSE           TNEYMDMKPGVSYVVPTKTDKRRAARIGSYIERDVAPSIMEDDELALDLEDLLSFSYQVA 773
HUMAN           TNEYMDMKPGVSYVVPTKADKRRSVRIGSYIERDVTPAIMEDDELALDLEDLLSFSYQVA 777
MOUSE           SNEYMDMKPGVSYVVPTKTDKRRSARIDSYIERDVTPAIMEDDELALDLDDLLSFSYQVA 775
                        685-761 kinase insert domain       ><582-937 protein kinase domain

                                                                           ↓ A835V (mouse Kit^{W-n}, semidominant white spotting)
                                                        ↓ R816W (mouse Kit^{W-f}, semidominant white spotting)
                              ↓ D790G (mouse Kit^{W-42J}, dominant white)   ↓ V831M (mouse Kit^{W-41J}, semidominant white spotting)
                                  ↓ R796G (human piebaldism with sensineural deafness)
HORSE           KGMAFLASKNCIHRDLAARNILLTHGRITKICDFGLARDIKNDSNYVVKGNARLPVKWMA 833
HUMAN           KGMAFLASKNCIHRDLAARNILLTHGRITKICDFGLARDIKNDSNYVVKGNARLPVKWMA 837
MOUSE           KAMAFLASKNCIHRDLAARNILLTHGRITKICDFGLARDIRNDSNYVVKGNARLPVKWMA 835

                                    ↓ F856S (mouse Kit^{Wads}, semidominant white spotting)
                         ↓ T847P (human piebaldism)
HORSE           PESIFNCVYTFESDVWSYGIFLWELFSLGSSPYPGMPVDSKFYKMIKEGFRMLSPEHAPA 893
HUMAN           PESIFNCVYTFESDVWSYGIFLWELFSLGSSPYPGMPVDSKFYKMIKEGFRMLSPEHAPA 897
MOUSE           PESIFSCVYTFESDVWSYGIFLWELFSLGSSPYPGMPVDSKFYKMIKEGFRMVSPEHAPA 895

HORSE           EMYDIMKTCWDADPLKRPTFKQIVQLIEKQISDSTNHIYSNLANCSPRQENS-AVDHSVR 952
HUMAN           EMYDIMKTCWDADPLKRPTFKQIVQLIEKQISESTNHIYSNLANCSPNRQKP-VVDHSVR 956
MOUSE           EMYDVMKTCWDADPLKRPTFKQVVQLIEKQISDSTKHIYSNLANCNPNPENPVVVDHSVR 955

HORSE           INSVGSSASSTQPLLVHEDV 972
HUMAN           INSVGSTASSSQPLLVHDDV 976
MOUSE           VNSVGSSASSTQPLLVHEDA 975
```
