# Supplementary material for: Allelic Heterogeneity at the Equine KIT Locus in Dominant White (W) Horses
Source: PLoS Genet. 2007 Nov 9;3(11):e195. doi: 10.1371/journal.pgen.0030195 (PMC2065884; doi:10.1371/journal.pgen.0030195)
Supplement: Supplementary file 3 [file pgen.0030195.st001.pdf]

Table S1. Genotype Distributions at *KIT* Polymorphisms

	White FM (n=20)[a]			Solid-c. FM (n=112)[a]			White Tb (n=8)[a]			Solid-c. Tb (n=66)[a]			White Ar (n=3)[a]			Solid-c. Ar (n=23)[a]			CW (n=6)[a]		
	TT	TG	GG	TT	TG	GG	TT	TG	GG	TT	TG	GG	TT	TG	GG	TT	TG	GG	TT	TG	GG
c.-87T>G	1	3	-	50	49	13	5	3	-	-	-	-	-	2	-	1	-	-	3	-	-
	AA	AT	TT	AA	AT	TT	AA	AT	TT	AA	AT	TT	AA	AT	TT	AA	AT	TT	AA	AT	TT
**c.706A>T**	**4**	-	-	**110**	-	-	**8**	-	-	**16**	-	-	-	**3**	-	**23**	-	-	**3**	-	-
	CC	CT	TT	CC	CT	TT	CC	CT	TT	CC	CT	TT	CC	CT	TT	CC	CT	TT	CC	CT	TT
**c.1805C>T**	**4**	-	-	**94**	-	-	**8**	-	-	**66**	-	-	**2**	-	-	**23**	-	-	-	**6**	-
	CC	CT	TT	CC	CT	TT	CC	CT	TT	CC	CT	TT	CC	CT	TT	CC	CT	TT	CC	CT	TT
c.1867+19C>T	2	2	-	79	15	-	7	1	-	-	-	-	2	-	-	1	-	-	3	-	-
	GG	GA	AA	GG	GA	AA	GG	GA	AA	GG	GA	AA	GG	GA	AA	GG	GA	AA	GG	GA	AA
**c.1960G>A**	**4**	-	-	**97**	-	-	**5**	**5**[b]	-	**17**	-	-	**2**	-	-	**1**	-	-	**3**	-	-
	AA	AG	GG	AA	AG	GG	AA	AG	GG	AA	AG	GG	AA	AG	GG	AA	AG	GG	AA	AG	GG
c.2045A>G	-	1	3	9	25	63	-	5	3	-	-	-	-	-	3	5	1	-		1	2
	GG	GA	AA	GG	GA	AA	GG	GA	AA	GG	GA	AA	GG	GA	AA	GG	GA	AA	GG	GA	AA
c.2100G>A	3	1	-	47	44	6	7	1	-	-	-	-	-	2	-	6	1	-	3	-	-
	CC	CG	GG	CC	CG	GG	CC	CG	GG	CC	CG	GG	CC	CG	GG	CC	CG	GG	CC	CG	GG
**c.2151C>G**	-	**20**	-	**112**	-	-	**8**	-	-	**46**	-	-	**2**	-	-	**26**	-	-	**3**	-	-
	GG	GA	AA	GG	GA	AA	GG	GA	AA	GG	GA	AA	GG	GA	AA	GG	GA	AA	GG	GA	AA
c.2244G>A	-	4	-	79	16	-	-	8	-	-	-	-	-	2	-	5	1	1	-	3	-
	AA	AG	GG	AA	AG	GG	AA	AG	GG	AA	AG	GG	AA	AG	GG	AA	AG	GG	AA	AG	GG
c.2472+11A>G	2	2	-	93	17	-	7	1	-	-	-	-	2	-	-	6	-	1	3	-	-
	AA	AG	GG	AA	AG	GG	AA	AG	GG	AA	AG	GG	AA	AG	GG	AA	AG	GG	AA	AG	GG
c.2472+181A>G	2	2	-	93	17	-	7	1	-	-	-	-	2	-	-	6	-	1	3	-	-
	AA	AG	GG	AA	AG	GG	AA	AG	GG	AA	AG	GG	AA	AG	GG	AA	AG	GG	AA	AG	GG
c.2473-37A>G	2	2	-	88	17	-	-	-	-	-	-	-	-	-	-	5	-	1	-	-	-
	AA	AG	GG	AA	AG	GG	AA	AG	GG	AA	AG	GG	AA	AG	GG	AA	AG	GG	AA	AG	GG
c.2685-36A>G	1	3	-	78	25	6	8	-	-	-	-	-	2	-	-	5	2	-	2	1	-
	CC	CT	TT	CC	CT	TT	CC	CT	TT	CC	CT	TT	CC	CT	TT	CC	CT	TT	CC	CT	TT
c.2739C>T	2	2	-	90	19	-	-	8	-	-	-	-	-	2	-	5	1	-	-	3	-
	CC	CG	GG	CC	CG	GG	CC	CG	GG	CC	CG	GG	CC	CG	GG	CC	CG	GG	CC	CG	GG
c.2791-28C>G	-	1	3	58	46	8	-	8	-	-	-	-	-	2	-	2	4	1	-	3	-
	CC	CT	TT	CC	CT	TT	CC	CT	TT	CC	CT	TT	CC	CT	TT	CC	CT	TT	CC	CT	TT
c.*136C>T	-	4	-	80	17	-	8	-	-	-	-	-	2	-	-	5	2	-	3	-	-
	GG	GA	AA	GG	GA	AA	GG	GA	AA	GG	GA	AA	GG	GA	AA	GG	GA	AA	GG	GA	AA
c.*252G>A	-	4	-	32	9	-	8	-	-	-	-	-	2	-	-	5	2	-	3	-	-
	CC	CT	TT	CC	CT	TT	CC	CT	TT	CC	CT	TT	CC	CT	TT	CC	CT	TT	CC	CT	TT
c.*285C>T	1	3	-	23	3	-	-	-	-	-	-	-	-	-	-	5	-	1	-	-	-

[a] Not all animals were genotyped for all polymorphisms. FM, Franches-Montagnes; Tb, Thoroughbred; Ar, Arabian; CW, Camarillo White Horse;

[b] The five white Thoroughbreds carrying the c.1960G>A mutation all belonged to one family. The other five white Thoroughbreds were not closely related to these animals.
