# Supplementary material for: Allelic Heterogeneity at the Equine KIT Locus in Dominant White (W) Horses
Source: PLoS Genet. 2007 Nov 9;3(11):e195. doi: 10.1371/journal.pgen.0030195 (PMC2065884; doi:10.1371/journal.pgen.0030195)
Supplement: Supplementary file 4 [file pgen.0030195.st002.pdf]

Table S2. Primer Sequences for the Amplification of equine *KIT* Exons and cDNA Fragments

Exon	Forward primer	Sequence	Reverse primer	Sequence	Product (bp)	Tm (°C)
1	KIT_Ex1_IF	TAGCACGTCGAAAGAGCTG	KIT_Ex1_IR	CTTCCCGGAGGCTGAAAC	433	58
2	KIT_Ex2_IF	AAATAGGGCAGCTTTGTCC	KIT_Ex2_IR	TATCAGAGCCTTGCAGACC	610	58
3	KIT_Ex3_IF	GAGTCAGCAGCCATTTGAG	KIT_Ex3_IR	CCTAGCTGGAGGCAGTCAC	610	60
4	KIT_Ex4_IF	GGATGCGTGATTTAATTGC	KIT_Ex4_IR	CACACCAGCTGAGCCATC	439	60
5	KIT_Ex5_IF	TGACAGACTTGTCATGATGC	KIT_Ex5_R	GCTGCGTAAACAACGTCAG	577	58
6	KIT_Ex6_F	TCCTGGGTCTGGGTATAGG	KIT_Ex6_R	TTGAAGGCACTCACTCTCC	869	60
7	KIT_Ex7_F	CGAAGGACCCTAGGAAGAA	KIT_Ex7_R	GGTAGCACCCAGAATGTGA	878	58
8	KIT_Ex8_F	TCACCTCTTCACCCCTCTC	KIT_Ex8_R	TGGAAATTGCACGATAAATCC	896	58
9	KIT_Ex9_F	TGCTTTGTACATCCTCTTGC	KIT_Ex9_R	GTGCATGGACAGAACACAC	888	58
10-11	KIT_Ex10_11_IF	GCTGTGAGATGGGAGGTG	KIT_Ex10_11_IR	CAAAGCTATCAAAGGTGGTG	569	58
12-13	KIT_Ex12_13_IF	CACCACCTTTGATAGCTTTG	KIT_Ex12_13_IR	TTTGGATGCAACATACGTG	694	58
14	KIT_Ex14_IF	CGGGTTTTGATAAGCAATG	KIT_Ex14_IR	CCACGATGAGAGAAACAGG	614	58
15	KIT_Ex15_IF	AAAGGCCATCTAGCTCCTG	KIT_Ex15_IR	CAACCCTTAACTGCCATTG	462	58
16	KIT_Ex16_IF	TTCACGTAGGGTCTCATGG	KIT_Ex16_IR	CCAAAGAGACAGCAGTTGG	671	58
17	KIT_Ex17_IF	TGGAGTTGGTTTTGAAAGTG	KIT_Ex17_IR	ACGTGCCCCATAATTACAC	598	58
18-19	KIT_Ex18_19_IF	TCATCTGAGGCCATACAGG	KIT_Ex18_19_IR	AAGGGCCATTTAGCATCAC	830	56
20	KIT_Ex20_IF	AAGGGCCAAGATGTGTTTC	KIT_Ex20_IR	GCCCAATTTATAGCCCAAG	588	58
21	KIT_Ex21_IF	GCTGTTGGGTTGTAGTTGG	KIT_Ex21_IR	CAACCATCCTTTTGGACAG	785	58
cDNA	KIT_MF	GTGAATCTTCTCGGAGCATG	KIT_MR1	GCAAGGAACGCCATGCCC	392	58
			KIT_MR2	CTATGAATACAGTTCTTTGAGG	413	58
